# Development of targeted strategies to enhance linkage and retention in HIV care among people living with HIV/AIDS in Mpumalanga province

**DOI:** 10.3389/frhs.2026.1929158

**Published:** 2026-09-03

**Authors:** Thuso Charity Mafhungo, Mygirl Lowane, Linda Skaal

**Affiliations:** Department of Public Health, Sefako Makgatho Health Sciences University, Pretoria, South Africa

**Keywords:** art, HIV, linkage to care, retention in care, strategies

## Abstract

**Background:**

Despite significant progress in HIV services, significant patient loss occurs at every stage of the HIV care continuum. While efforts made by the NDoH aim to combat the HIV epidemic and achieve the 95-95-95 targets, these targets cannot be reached unless PLHIV are effectively linked to and retained in care. This study aims to develop targeted strategies to improve linkage to and retention in HIV care among PLHIV in the Msukaligwa Sub-district, Mpumalanga Province.

**Methods:**

A convergent parallel mixed-methods design will be employed. The study will consist of four phases: (1) a scoping review to identify global strategies for improving HIV care outcomes; (2) a quantitative strand using descriptive cross-sectional and retrospective cohort designs with TIER.Net data (2020–2024) to assess linkage and retention patterns; (3) a qualitative exploratory study using semi-structured interviews with PLHIV and healthcare providers to explore lived experiences, barriers, and facilitators; and (4) development of an evidence-based, context-specific strategy. Quantitative data will be analysed using descriptive and inferential statistics, while qualitative data will undergo thematic analysis guided by the Socio-Ecological Model.

**Expected results:**

The study is expected to identify key individual, interpersonal, and health system factors influencing poor linkage and retention in HIV care. It will also highlight critical gaps along the HIV care continuum and provide insight into patient and provider perspectives.

**Conclusion:**

Findings from this study will inform the development of a tailored, evidence-based strategy to strengthen HIV care engagement in the Msukaligwa Sub-district. This may contribute to improved retention, viral suppression, and progress toward national and global HIV targets.

## Introduction

In 2024 an estimated 40.8 million people were living with Human Immunodeficiency Virus (HIV) globally, of which 1.4 million were children 0–14 years old and 39.4 were adults 15 years and above (1). HIV incidence was approximately 1.3 million, with children accounting for 120,000 of the new infections in 2024 (2). Since the beginning of the epidemic, it has been reported that 91.4 million people have acquired HIV globally. HIV has claimed 44.1 million lives globally as of 2022 since its identification in 1983 (3). This number is alarming, and unaddressed HIV could progress to global health catastrophe. Since 2014, the Joint United Nations Program against HIV/AIDS set the 90-90-90 targets aiming to diagnose 90% of People Living with HIV (PLHIV), initiate Antiretroviral Therapy (ART) in 90% of those diagnosed, and achieve viral suppression in 90% of those on treatment by 2020. These goals were later expanded to 95-95-95 by 2030 (4). Despite progress, many countries still face challenges in achieving these targets (5).

Globally 87% of PLHIV knew their status, 77% were accessing treatment and 73% were virally suppressed in 2024 (2). It is evident that South Africa is doing well in its testing interventions, there are however substantial gaps between the 2nd 95 and the 3rd 95 target. This can either be because patients are not being linked to care after being diagnosed with HIV for ART initiation or they are lost after they have been initiated on ART. According to the district health barometer 2023/24 Mpumalanga recorded a 75.2% ART retention rate, with 93% of retained patients achieving viral suppression while Gert Sibande district recorded 80% and 93% viral load suppression (6).

Linkage to and retention in care are critical components of the HIV care continuum. This continuum serves as a vital public health model, outlining the sequential stages that patients with HIV progress through from diagnosis to achieving and sustaining viral load suppression through the utilization of ART (4). These stages include the following: HIV diagnosed, linkage to care, initiated to treatment, retained in care and achieving and maintaining viral load suppression to undetectable levels (7).

There are several efforts that the South African National Department of Health (NDoH) has made to tackle the quality of care offered to PLHIV. These efforts include the national wellness campaign known as “Cheka Impilo” which aims to strengthens HIV prevention strategies and linkage to care; the operation phuthuma project which focusing on prioritization of high-volume facilities and implements a ten-point acceleration plan to enhance service delivery; as well as the welcome back campaign which provides strategic guidance and support to ensure effective re-engagement of patients who have disengaged or who are lost to follow up from HIV care for different reasons (8).

On the 25th of February 2025, the Minister of Health, Dr Aaron Motsoaledi launched the closing 1.1 million HIV treatment gap campaign to fast-track progress to reach the 95-95-95 UNAIDS HIV targets (9). The main objective of this campaign was to identify and support PLHIV who are not initiated on ART and those who are lost in care (10). In 2016 NDoH introduced and implement the UTT policy, by which all HIV diagnosed patients must be initiated on ART irrespective of CD4 count (11). The prevention access campaign launched the Undetectable = Untransmittable (U = U). According to this strategy PLHIV who are on ART and virally suppressed has a zero risk of transmitting HIV sexually (12).

The Sustainable Development Goal three aim to end the HIV/AIDS epidemic by 2030 (13). Achievement of this goal will be reached when the number of new HIV infections and AIDS-related deaths has been declined by 90% between 2010 and 2030 (28). HIV pandemic is not progressing toward an end, if corrective measures are not implemented, it may resurge and is expected to remain a significant global health challenge for the foreseeable future (14).

Progress toward the 2030 HIV targets depends on linkage and retention strategies that integrate quality care, respect for patient choice, and empowered patients who are treatment literate and supported to manage their own treatment effectively (15). Globally HIV programmes and stakeholders are now acknowledging that at some point PLHIV will be disengaged from ART as their preferences, needs, and behaviours change. Health systems must be prepared to identify and prepare for this disengagement and prioritizing on reducing the frequency and duration of disengagement (5).

Despite progress in HIV services, significant patient loss occurs at every stage of the HIV care continuum (16). While efforts made by the NDoH aim to combat the HIV epidemic and achieve the 95-95-95 targets, these targets cannot be reached unless PLHIV are effectively linked to and retained in care, therefore the researcher seek to address this gap by exploring factors influencing linkage and retention in HIV care and to develop targeted strategies to improve patient outcomes and support national HIV goals.

The aim of the study to develop targeted strategies to improve linkage and retention in HIV Care among PLHIV in Mpumalanga Province. The study will undergo four phases:.

## Theoretical and conceptual frameworks

This study will be informed by two complementary frameworks: the Socio-Ecological Model (SEM) as the theoretical framework and the HIV continuum of care as the conceptual framework. Urie Bronfenbrenner in the 1970s developed the SEM, which defines that human behaviour is influenced by interactions through many stages of influence: individual, interpersonal, community, organizational, and policy levels (17, 18). The SME will aid as a roadmap in the qualitative strand. SME offers a theoretical foundation for grasping how human behaviour is influenced by the interaction of many stages of influence, these will allow the researcher to explore factors affecting linkage to and retention in HIV care from all the five stages.

The HIV Continuum of Care outlines the sequential stages in HIV care: diagnosis, linkage to care, initiation of ART, retention in care, and achievement of viral suppression (19). It will be employed in this study to frame the quantitative component, where secondary data from TIER.Net will be analysed to assess where patients are lost across the care cascade. This framework will aid in identifying critical stages where patients are most likely to drop out of care. Analysing trends and factors associated with poor outcomes along the HIV continuum of care helps in identifying strategic opportunities for targeted interventions to strengthen linkage to and retention in HIV care.

## Framework integration and application

By integrating the SEM and the HIV continuum of care, this study integrates behavioural, systemic, and service delivery perspective, the continuum of care maps the patient's clinical pathway, on the other hand the SEM allows for a deeper understanding of why patients may fail to transition between stages. This dual framework approach will be implemented across the three phases of the study:

### Quantitative strand

The HIV continuum of care will be utilized to assess linkage and retention patterns in TIER.Net data.

### Qualitative strand

Since human behaviour is influenced by the interaction of many stages of influence, the SME will aid in exploring factors affecting linkage to and retention in HIV care.

### Strategy development

The strategy will be developed from the findings from both frameworks.

## Research methodology

### Research design

Convergent parallel mixed methods design will be employed in this proposed study to explore factors influencing linkage to and retention in HIV care. In this methods qualitative and quantitative data will be collected concurrently and analysed independently, then the findings will be merged and interpreted together.

### Study setting

The proposed study will be conducted in one regional hospital and ten primary health care facilities (PHC) situated in Msukaligwa Sub-district, Gert Sibande district Mpumalanga Province in South Africa. Mpumalanga is neighbouring Swaziland and Mozambique, is in the eastern part of the country. There are three districts in Mpumalanga province: Gert Sibande, Ehlanzeni, and Nkangala, and 18 local municipalities. The Gert Sibande district is made up of the following sub district: Chief Albert Luthuli, Pixley Ka Isaka Seme, Mkhondo, Lekwa, Dipaliseng, Msukaligwa, and Govan Mbeki Local Municipalities (20). From the seven municipalities in the district Msukaligwa is the largest, accounting for 19% of the district's total geographical area. The municipality is located in Ermelo and delivers services through 10 PHC facilities and one regional hospital (21).

### Population

People living with HIV who are accessing and have accessed care at PHC facilities and Ermelo regional hospital and healthcare workers in Msukaligwa Sub-district of the Gert Sibande District, Mpumalanga Province, South Africa.

### Inclusion criteria

#### Phase 2

- Patients diagnosed with HIV between January 2020 and December 2025.
- Patients who are accessing HIV care in any of the PHC facilities and in Ermelo hospital within the Msukaligwa Sub-district.
- Records with complete data on demographic information, ART initiation, and follow-up visits.

#### Phase 3

- PLHIV who are on ART in Msukaligwa sub district
- Healthcare providers involved in HIV care with at least one year of experience
- PLHIV 18 years of age and older

### Exclusion criteria

- Records with missing or incomplete critical variables.
- HIV positive patients with mental health conditions

### Phase one: scooping review

Scooping review will be conducted to map the existing literature on the strategies used to improve HIV outcomes care globally. Scoping review will be conducted based on the framework developed by Arksey and O'Malley, which will be implemented through the following six step: Identifying the research question, identifying relevant studies, study selection, charting the data and collating, summarising and reporting the results (22). The Prisma-ScR flow diagram will be utilized to demonstrate the process of the study selection.

### Phase two: quantitative strand

#### Study design

This strand will employ two complementary designs: a descriptive cross-sectional and a retrospective cohort design. Descriptive cross-sectional design will be utilized to evaluate the status of linkage and retention in HIV care among PLHIV in Msukaligwa sub district. This design will allow the researcher to determine the proportion of patients who are linked to care after diagnosis and those who are retained in care over time. The study will further highlight the prevalence of missed linkage, loss to follow-up, and late ART initiation and this will be characterized by age and sex, offering insight into which groups are mainly affected by gaps in HIV care.

A retrospective cohort design will be employed to track and analyse trends in linkage to and retention to HIV care for the period 2020–2024. Using routinely collected from TIER.net data, this design will facilitate the study to examine trends over time in linkage to care, retention in care, ART initiation, and follow-up outcomes within patients diagnosed with HIV during the study period.

#### Sampling methods

The study will conduct a multistage sampling where: for the descriptive cross-sectional design, a systematic random sampling and for the retrospective study case in the TIER.Net data base from January 2020 to December 2024 will be included.

#### Sample size

The sample size for this study will be calculated using OpenEpi version 3.01. OpenEpi is an open-source epidemiologic calculator, based on the estimated prevalence of 50%, a 95% confidence level, and a 5% margin of error and an estimated population of 5000. The population that will be used will be taken from the current total remaining on ART (TROA) from Tier.Net during the study period. The sample size will then be divided according to the 11 healthcare facilities in Msukaligwa sub district. The distribution will be proportional to the healthcare facility patient population size which will be the current TROA during the study period.

#### Data collection tools

For the descriptive cross-sectional study, a structured survey questionnaire will be constructed to collect data from PLHIV receiving care in healthcare facilities in Msukaligwa sub district. The variables on the questionnaire will include socio-demographic, clinical information, and factors influencing linkage and retention in HIV care. To ensure simplicity of analysing results and to ensure standardization within the selected participants closed-ended questions will be used. To ensure reliability, the questionnaire will be piloted in a subset of participants with similar characteristics to the targeted study population, but they will not form part of the main study. Input gathered during piloting of the questioner will be utilized to refine the wording, clarity, and sequence of the questions. Test-retest reliability will be assessed during the pilot to determine the stability of responses over time. Internal consistency will be checked for questions in the interview guide that measure the same idea, like barriers to HIV care. Cronbach's alpha will be used to see if the questions are related.

Content validity will be ensured by designing interview guides and data collection tools based on an in-depth literature review and theoretical frameworks such as the Socio-Ecological Model. This will help guarantee that all relevant individual, social, and health system factors influencing linkage and retention are thoroughly explored. Face validity will be ensured by reviewing interview questions and quantitative indicators by the supervisor and the HIV expect to ensure they clearly address the key aspects of the study, such as barriers to linkage, retention, and engagement in HIV care. In this study, validity will be ensured by aligning interview questions and quantitative indicators with the following well-established theoretical frameworks the Socio-Ecological Model and the HIV continuum of care. While criterion validity will be established by comparing participants' self-reported behaviors (e.g., appointment attendance, ART adherence) with objective data from TIER.net. This cross-check will help confirm that interview responses align with actual clinical records, ensuring that the measures accurately reflect real-world outcomes. For retrospective study data will be extracted from Three Interlinked Electronic register (TIER.net).

TIER.Net is a non-networked electronic programme which captures TB/HIV patient information. A structured data extraction template will be utilized to collect data. Data will be recorded and analysed using Microsoft Excel. The template will include the following variables:

- Demographics: Age, date of birth and sex
- HIV Diagnosis Date: To establish the point of entry into the HIV care continuum.
- ART Initiation Date: To measure linkage to care
- Outcomes: To assess patient treatment outcome
- Viral Load Results and Dates: To determine viral load suppression rate

#### Data collection procedure

For survey, data collection will be conducted in the health facilities, the researcher will distribute surveys to PLHIV who are present at the healthcare facility during the dates of data collection. The sample will be selected using systematic random sampling, targeting participants who meet the inclusion criteria. Informed consent will be obtained from all participants, ensuring that they are well informed about the study purpose, their voluntary participation, and that their responses will be kept confidentially.

For retrospective study data extraction will be conducted by the researcher. The researcher will work in closely with facility data capturers and sub district information officer to ensure the accuracy and completeness of the extracted information. To comply with ethical standards and protection of patient confidentiality only de-identified data will be exported for analysis. The researcher will obtain formal approval to use the TIER.net database from the Mpumalanga Provincial Department of Health research committee and the Gert Sibande District Health Office before data collection. Data will be collected using a structured extraction template, aligned with the study objectives and the HIV care continuum conceptual framework. Data protection will be maintained by ensuring that all extracted data are stored in a password protected computer, and access will be limited to the researcher. External hard drive will be used for data back-up it will be stored in a locked cabinet to prevent unauthorized access or data loss.

#### Data analysis plan

Quantitative data will be analysed using descriptive and inferential statistical methods. Collected data will be assessed for data quality and validation to check for missing values, and inconsistencies. Descriptive statistics such as frequencies, percentages and means will be used to summarize socio-demographic characteristics, and patterns of healthcare engagement among participants. Associations between independent variables and key HIV care outcomes: timely linkage to care and retention in HIV care will be analysed using inferential statistics. Relationships between categorical variables and outcomes will be assessed using Chi-square tests. Independent *t*-tests will compare mean values of continuous variables across outcome groups. Odds ratios (ORs) with 95% confidence intervals (CIs), will be calculated to quantify association and statistical significance will be considered at *p* < 0.05. Data will be analysed using Microsoft Excel software and Stata statistical software, and results will be presented in tables and graphs.

#### Reliability and validity

Reliability will be ensured through Test-Retest Reliability and Internal consistency. Validity will be established through criterion, construct, content and face validity.

### Phase three: qualitative strand

#### Study design

A qualitative descriptive exploratory study design will be conducted to explore the lived experiences, perceptions, and challenges related to HIV linkage and retention among PLHIV and healthcare providers in Msukaligwa sub-district. All 11 healthcare facilities in the Msukaligwa Sub-district will be included in the qualitative strand of the study. Purposive sampling, which is a non-probability sampling technique, will be employed to select participants who meet the study inclusion criteria. The estimated sample size of 20–25 PLHIV will be proportionally distributed among the 11 healthcare facilities based on each facility's Total Remaining on ART (TROA). This approach will ensure that the number of participants selected from each facility reflects the proportion of PLHIV receiving ART services at that facility, while ensuring representation of all selected healthcare facilities in the study.

During the data collection period, eligible PLHIV attending the selected healthcare facilities will be identified according to the predefined inclusion criteria. Participants who meet the eligibility requirements will then be purposively selected from each. Only PLHIV, who are 18 years and older and currently receiving ART services within the Msukaligwa Sub-district will be eligible to participate. Recruitment will continue until the required sample size of 20–25 PLHIV is achieved and data saturation is reached. Similarly, 15–20 healthcare providers, including nurses and HIV lay counsellors involved in HIV care services, will be purposively selected from the 11 healthcare facilities. The selection of healthcare providers will be based on predefined inclusion criteria, including being directly involved in HIV care services and having at least one year of experience in HIV service delivery.

#### Data collection tools

Semi-structured interview guide will be constructed for the PLHIV and healthcare workers. This tool will explore: Personal experiences with HIV diagnosis and care linkage, barriers and facilitators to continuing care, perceived health system and structural challenges, and support mechanisms and suggestions for improvement.

#### Data collection procedure and recruitment

Participants will be recruited purposively from among patients attending Ermelo hospital and PHC facilities in the Msukaligwa Sub-district. Eligible participants will be provided with a Participant Information Sheet explaining the purpose and objectives of the study, the procedures involved, expected duration of participation, potential risks and benefits, confidentiality and data protection measures, voluntary nature of participation, and their right to withdraw from the study at any stage without providing a reason and without any effect on the care they receive. If they agree to participate in the study the researcher will explain the purpose of the study, and that participation is voluntary, and that participants may withdraw at any stage without providing reason and it won't affect their care. The researcher will provide an opportunity for participants to ask questions and clarify any concerns before obtaining informed consent. Written informed consent for participation and for audio record during the interview will be obtained.

A semi-structured interview guide will be utilized to explore participants' experiences with HIV care, including linkage to treatment, retention, challenges faced, and their perceptions of the healthcare system. Interviews will be conducted by the researcher in the participant's preferred language. With informed consent, all interviews will be audio-recorded, transcribed verbatim, and translated into English where necessary. Field notes will be taken to capture contextual details and non-verbal cues that may enrich the data. Data will be collected until saturation where there will be no new information surfaced.

#### Data analysis plan

The qualitative data will be analysed by a hybrid thematic analysis that incorporate deductive and inductive strategies. Deductive coding will be guided by the SEM, offering a structured framework to explore barriers and facilitators to linkage and retention in HIV care across multiple levels, namely individual, interpersonal, health system, and structural contexts. Inductively, the researcher will allow for new and unexpected themes to emerge organically from participants' narratives to ensure that the analysis remains grounded in the lived experiences of the study population. All interviews will be transcribed verbatim and translated into English when necessary. Coding will be done either manually or using qualitative data analysis software, based on the availability of the resources.

Trustworthiness will be ensured through credibility, dependability, confirmability: transferability. Bias will be minimized by addressing selection response researcher publication and analytical bias.

## Phase four: development of strategy

The development of the proposed strategy will be guided by the Intervention Mapping (IM) framework. IM is a framework which is informed by conceptual and evidence-based decision making, collaborative design and participatory-based research techniques (23). It was designed to provide a framework on the development of evidence-based behavioral strategies in the health context. IM comprises six systematic steps that facilitate the integration of theory, evidence, and stakeholder collaboration in collaboratively designing interventions to address identified health problems (24).

### Step 1: needs and assets assessment

The findings from quantitative and qualitative components will assist in identifying barriers, facilitators and gaps that influence HIV linkage and retention in care.

### Step 2: intervention outcomes and objective

The identified barriers, facilitators and gaps will aid in formulating objectives aimed at improving HIV linkage and retention in care.

### Step 3: selection of evidence-based strategies

The results from scooping review will be utilized to identify strategies that other countries have implemented and which ones were effective in improving linkage to and retention to HIV care.

### Step 4: strategy development

The researcher will therefore compile a comprehensive report based on the findings from quantitative, qualitative components and scooping review. The findings will be presented to Gert Sibande District managers to obtain their input and collaboratively develop the strategy. This participatory process will ensure that the final strategy is feasible, align with district priorities, and practical applicable in our study setting.

### Step 5: implementation mapping

The district managers will give their input based on the strategies from scooping review and select the strategy that seems feasible and align with our context and give input on the process of implementing the strategy.

### Step 6: evaluation plan

The strategy will be piloted in selected healthcare facilities within the Msukaligwa Sub-District to assess its effectiveness in improving linkage and retention outcomes and its usefulness, acceptability, and feasibility within routine healthcare settings. Input from the pilot phase will be used to modify the strategy before its implementation.

### Ethical considerations

The study was reviewed by the Department of Public Health Research Committee and subsequently submitted to the School of Healthcare Sciences Research Committee (SHCSRC). The SHCSRC recommended that the study be submitted to the Sefako Makgatho Health Sciences University Research Ethics Committee (SMUREC) after incorporating the revisions suggested during the review process. The recommended revisions were addressed, and the study was subsequently submitted to SMUREC, where it is currently awaiting ethical clearance. Upon approval, the ethical clearance and research proposal will be submitted to the Mpumalanga Provincial Research Committee in Nelspruit for further review and approval to conduct the study at the healthcare facilities in Msukaligwa sub district. After obtaining approval from the provincial committee, the researcher will submit the provincial approval letter to the Msukaligwa sub-district office to proceed with the study. Patient confidentiality will be maintained throughout the study, and ethical principles of respect for persons, beneficence, justice and autonomy will be observed during the consenting process.

### Confidentiality and anonymity

Due to the sensitive nature of HIV status, rigorous measures will be implemented to protect the identity and confidentiality of PLHIV participating in the study. The researcher will utilize codes rather than participant's name to assure participants anonymity. All data collected from participants will be securely stored in password-protected files accessible only to the researcher and the supervisor. Hard-copy documents, such as questionnaires and consent forms, will be stored in a locked cabinet in a secure location. Personal identifiers will be excluded from reports or publications resulting from the study. For retrospective TIER.Net data, only de-identified information required for the study objectives will be extracted, with all direct identifiers removed before analysis.

## Dissemination of study findings

The findings of this study will be disseminated through a written research protocol and academic publications. In addition, the results will be presented to key stakeholders, including the Gert Sibande District Primary Health Care (PHC) programme managers and the operational managers for the Msukaligwa Sub-district. These dissemination meetings will provide an opportunity to share the study findings, obtain stakeholder input, and engage in collaborative discussions to inform the development of context-specific strategies aimed at improving linkage to and retention in HIV care. This approach will ensure that the study findings are translated into practical and actionable interventions within the district health system.

## Discussion

This study seeks to address gaps in linkage to and retention in HIV care, which remain critical barriers to achieving optimal HIV treatment outcomes and the 95-95-95 targets. The findings of this study are expected to strengthen existing evidence that disengagement from care occurs across multiple levels, including individual, interpersonal, and health system factors. Using the Socio-Ecological Model (SEM) as the theoretical framework the study will be able to explore the individual level, factors such as stigma, fear of disclosure, treatment fatigue, and socioeconomic challenges are likely to influence patients' ability to remain engaged in care (17, 18). Most PLHIV start and stop ART several times in their lives, some patients fear being judged or mistreated when returning to care after missing appointment dates (5). The qualitative component of this study is expected to provide deeper insight into these lived experiences and how they shape care-seeking behaviours.

At the interpersonal and community levels, social support systems, family dynamics, and community perceptions of HIV play a significant role in influencing retention. Perger et al. (25) mentioned that lack of support or fear of discrimination may discourage individuals from attending clinics or adhering to treatment. Conversely, strong support networks and community-based interventions may enhance retention and adherence. Health system factors are also expected to emerge as key determinants of linkage and retention. Challenges such as long waiting times, negative attitudes from healthcare providers, poor patient-provider communication, and inefficient follow-up systems can contribute to disengagement from care (26, 27). While national initiatives such as Universal Test and Treat (UTT) and re-engagement campaigns have improved access, their effectiveness depends on consistent implementation at facility level.

By integrating the Socio-Ecological Model and the HIV care continuum, this study provides a comprehensive understanding of where and why of patient loss at different stages of the continuum of care. The quantitative findings will identify critical points of retention loss, while qualitative insights will explain the underlying reasons for these gaps. This combined approach strengthens the validity of the findings and ensures that the proposed strategies are both evidence-based and contextually relevant. The development of a tailored strategy in collaboration with district managers and programme coordinators is a key strength of this proposed study. This collaborative approach increases the likelihood of feasibility, acceptability, and sustainability of the strategy. The pilot testing of the strategy will further allow for refinement before implementation. Overall, this study is expected to contribute valuable evidence to inform targeted interventions aimed at improving linkage to and retention in HIV care. Strengthening these components is essential for improving patient outcomes, achieving viral suppression, and ultimately reducing HIV-related morbidity and transmission in the Mpumalanga Province and similar settings.

## Conclusion

- First study of its kind in Msukaligwa Sub-district on HIV linkage and retention
- Findings will inform a targeted strategy to improve HIV care outcomes
- It uses a mixed-methods approach combining quantitative and qualitative data.
- Multi-level factors influencing care engagement will be examined
- The study is limited to one sub-district, which may reduce generalisability to other districts or provinces
